# Assessing the multiple benefits of partially protected marine protected areas in Australia: A systematic review protocol

**DOI:** 10.1371/journal.pone.0284711

**Published:** 2023-04-20

**Authors:** Genevieve A. C. Phillips, Nils Krueck, Emily Ogier, Neville Barrett, Ian Dutton, Klaas Hartmann

**Affiliations:** 1 Institute for Marine and Antarctic Studies, The University of Tasmania, Taroona, Tasmania, Australia; 2 Centre for Marine Socioecology, The University of Tasmania, Hobart, Tasmania, Australia; 3 Department of Natural Resources and the Environment, Hobart, Tasmania, Australia; MARE – Marine and Environmental Sciences Centre, PORTUGAL

## Abstract

**Background:**

There is global pressure to protect more of the world’s oceans, primarily to protect biodiversity, and to fulfill the “30 by 30” goal set by the International Union for the Conservation of Nature (IUCN) that has recently been ratified under the Kunming-Montreal Global Biodiversity Framework at the fifteenth Conference of Parties (COP-15). Fully protected marine protected areas (MPAs) provide the highest level of protection for biodiversity from destructive or extractive practices and may limit access to the area itself. Fully protected MPAs (also commonly referred to as ‘no-take MPAs’) ban all fishing activities, thereby removing the realisation of direct economic and social benefits from resource extraction within these areas. However, fully protected MPAs can still act as source of productivity to surrounding areas, while also providing an important scientific reference role for off-reserve management thereby providing indirect economic and social outcomes, as well as biodiversity benefits. Sustainable marine resource management strives to achieve ‘triple-bottom line’ benefits, where economic, social, and biodiversity benefits are maximised in managed areas of the ocean. Implementing ‘partially protected’ areas (PPAs) in areas of high biodiversity value (i.e., inshore, productive areas of the ocean) that allow for some extractive activities, may allow us to supplement fully MPAs to meet IUCN conservation goals, while maximising social and economic benefits. However, our current understanding lacks explicit quantitative assessments of whether and how PPAs can benefit (or otherwise) biodiversity, while also providing economic and social benefits. This study provides a method to systematically review the scientific and legislative literature to understand how PPAs may contribute to conserving biodiversity while also providing social and economic benefits to Australia.

**Methods and expected outputs:**

The implementation of partially protected areas (PPAs) requires careful consideration of many potentially competing factors, and an understanding of the types of partial protection already in place in a region. We have developed a systematic literature review protocol focussing on the primary research question: “What is the current state of partially protected area (PPA) implementation across Australian marine areas?”. The aim of the review is to provide marine resource managers with a comprehensive overview of PPAs in Australia, including associated goals and stated management strategies to achieve these goals, and a methodological approach that may be utilised globally.

The review protocol was designed by the research team for a Fisheries Resource and Development Corporation (FRDC) strategic research grant and will seek input from a project steering committee for the project on aggregation of the initial results. The steering committee is made up of stakeholders from a wide range of backgrounds and interests, covering marine conservation, fisheries management, Indigenous values, and academic research in Australia. Multiple academic databases, alongside Australian Federal, State, and Territory legislation and related policies will be reviewed using Boolean keyword search strings for both academic databases and relevant grey literature. Results from eligible documents will be compiled and insights from the review collated to provide information on the status of PPA implementation in Australia.

## Introduction

### Rationale

Protecting areas of natural habitat in the ocean by reducing or preventing destructive activities is of vital importance to protect biodiversity. The International Union for the Conservation of Nature (IUCN) has declared that 30% of our oceans (and freshwater areas, and land, respectively) need to be conserved or protected by 2030, based on scientific advice [1]. This has recently been ratified in the adoption of the Kunming-Montreal Global Biodiversity Framework at the fifteenth Conference of Parties (COP-15) in December 2022, where targets two and three relate to the conservation of 30% of degraded habitats are under effective restoration, and 30% of the earth are effectively conserved and managed (Kunming-Montreal Global Biodiversity Framework (GBF) [2]). Australia has recently announced its commitment to the “30 by 30” goals in response to the release of the marine chapter of the Australia State of the Environment report, released in July 2022 [3], and is a signatory of the Kunming-Montreal Global Biodiversity Framework. In practice, this means that at least 30% of Australia’s oceans should be protected from all forms of destructive activities, and that these protected areas should preferably be in regions of key importance to be effective at preserving (or at least maintaining) biodiversity.

To properly enact MPAs and ensure that relevant outcomes can be defined for specific areas, MPAs should first be defined appropriately according to their stage of establishment, level of protection, the measured outcomes, and the social and ecological conditions under which the MPA has been designated. The MPA Guide [4,5] has provided a novel and flexible classification framework that builds on IUCN Protected Area Categories for management objectives and governance types. The MPA Guide was chosen for this study as it simplifies the definitions set out by the IUCN and provides context around MPA effectiveness. For example, an MPA may be categorised *on paper* as an IUCN 1a MPA (no-take), however if there is poor park management in the region, or no clear management plan for the area, the park is unlikely to meet the criteria for an IUCN 1a MPA *in practice*. For the purposes of our review, and subsequent work in this area, we therefore use definitions as outlined in The MPA Guide.

Fully protected MPAs represent the highest level of protection from destructive activities. These areas of the ocean are primarily focused on conserving nature, as is the primary goal of MPAs [4,6]. Fully protected MPAs do not allow any fishing, mining, dredging, or dumping activities, and minimal other activities (see Fig 1 in reference [4]). The biodiversity benefits of fully protected MPAs are well documented [7–9], including positive impacts on fisheries both directly through spill-over effects [10] and indirectly via their reference area role in informing regional fishery management (e.g., [11]). Fully protected areas also have the potential to derive economic and social benefits simply by providing areas of restored and healthy ocean. Measuring and estimating the economic benefits of highly protected MPAs is complex due to a paucity of information on non-market economic benefits[12]. Fully protected MPAs may not meet their desired outcomes if not supported by local communities and stakeholders [13]. Consequently, there is growing global interest in implementing partial protection in the ocean, particularly in inshore areas where conflicts between multiple stakeholders are highest, and allowing multiple user groups access to resources could reduce conflict, while still providing biodiversity benefits. PPAs, for the purposes of this study, are MPAs that allow some form of extractive activity (usually fishing), while being managed as a ‘marine protected area’.

Recent studies within Australia are conflicted on the effects of PPAs. One study using data from baited remote underwater video stations (BRUVS) suggested PPAs may be an effective compromise between fully protected MPAs and areas of the ocean that do not have any formalised protection, particularly where resident species are important for commercial and recreational fisheries [14]. Another study in the same area of Australia found no ecological benefits of PPAs, and suggested that often the predicted social and economic benefits of PPAs are not realised and that PPAs merely create an illusion of protection, while consuming scarce public funds directed towards conservation [15]. Impacts on commercial fish species are also mixed–sometimes species benefit from PPAs, even as much as fully protected MPAs [16], whereas other studies have found no benefit to commercial species compared to open areas [17]. A recent global review of the use of PPAs suggests that these broad management tools can help balance biodiversity conservation, economic returns and social benefits, particularly where fully protected MPAs are practically infeasible and effective fisheries management is difficult to implement everywhere [18]). Current knowledge of the measurable effects of PPAs appears to be conflicting, often due to diverse methodologies and a lack of coherence in what should be measured, and when, to identify whether a MPA or PPA is effective. The systematic review proposed here aims to consolidate literature from diverse sources to provide a comprehensive overview on the use and potential or proposed benefits of marine PPAs, including suggestions on what to measure and when to measure it.

### Stakeholder engagement

The need for further evidence on the benefits of PPAs was recognised by the Fisheries Research and Development Corporation (FRDC) in their strategic research plan [@FRDC Strategic Research Plan 2020–25], and a call for research projects was initiated in August 2021. The work in this systematic review is part of a funded project by the FRDC (Project 2021–070) and will form the basis of the report for marine resource managers.

The project team, and authors of this systematic review, is made up of ecologists, fisheries scientists, social and economic scientists, and a natural resource manager. The project team established the scope of the review, relevant research questions and search terms (Tables 1 & 3) involved in determining eligibility criteria, contribute to reviewing literature, and assist in writing the manuscript.

**Table 1:** Timeline of project team and stakeholder consultation for the development of the systematic literature review protocol.

Meeting	Date	Participants	Topics discussed with stakeholders
**Scoping meeting**		Project team	Scope of literature review
			Primary research question
			Secondary research questions
			Boolean search terms
			Relevant databases
			Stakeholder involvement
			Protocol manuscript
**Email feedback**	28/07/22–28/08/22	Project team	Primary research question
			Secondary research questions
			Desired outputs
**Final protocol discussion (prior to submission)**	12/09/22	Project team	Final feedback on scope and design
			Discussion on protocol registration and publication
Resubmission meeting	27/02/23	Project team	Scope of literature review
			Primary research question
			Secondary research questions
			Boolean search terms
			Relevant databases
			Reviewer comments and letter to Editor
			Protocol manuscript–final edits to resubmission

The project has a Steering Committee comprised of members from fisheries management, fisheries research, conservation biology research, research scientists, and members of the Indigenous Community within Australia. The Steering Committee will be involved in ensuring the outputs from the literature review are in a format that is valuable and accessible not only for scientists but also marine resource managers and marine resource users.

### Objectives–research questions to answer

The systematic review seeks to address the following primary and secondary research questions in Australia.

#### Primary question

What is the current understanding of the effectiveness of partially protected areas (PPAs) in Australia?

### Secondary questions

1. How are PPAs defined across Australia?
2. What is the current state of PPA implementation across Australian marine areas?
  - How many PPAs are there?
  - What is the level of PPA coverage of Australia’s waters (% of ocean area and habitats)?
3. What legislation and policy defines PPAs in Australia?
4. How do definitions of PPAs in Australia map to The MPA Guide and the IUCN Guidelines?
5. What are the stated goals of PPAs in Australia?
6. Are stated goals of PPAs being addressed through defined operational instruments such as management strategies and programs or operational objectives?
7. How are the stated goals and objectives of PPAs monitored in Australia, considering:
  - biological (or biodiversity)
  - economic
  - social, and
  - cultural goals and objectives?
8. Do the stated goals define fair access to marine resources?
9. Are PPAs implemented primarily for conservation or fisheries resource sustainability objectives?
10. Are social and economic indicators incorporated into PPA implementation?
11. For PPAs that have been implemented for more than 10 years (i.e. 2011 onwards), have there been any major changes to management approaches?

## Methods

### Literature search strategy

The review will be based on several protocols for performing systematic literature reviews in the field of ecology [19–24]. A comprehensive list of potential databases and specialised websites, along with search terms including Boolean operators, was drawn up. To ensure a literature review was completed within practicable and feasible timeframes, search phrases were tested to ensure they produced relevant results without significant biases. This meant that search phrases did not include words that could bias results such as “benefits” or “disadvantages”. Two initial searches were conducted and refined to reduce the number of irrelevant publications (publications not related in any way to marine protected areas in Australia). The Boolean search are found below (Table 3).

The first round of databases for literature searches were chosen based on the Murdoch University Library recommended databases for systematic reviews in the following subject areas–“Australian Indigenous Studies”, “Biological Sciences”, “Environmental and Conservation Sciences”, “Sustainability”, “Global Politics and Policy”, and “Tourism and Events” (https://libguides.murdoch.edu.au/systematic/databases). The list was first reduced by whether the University of Tasmania had access to the databases, and then further by replicates of suggested databases. The final list of databases for the literature review was decided based on consultation with the whole research team and is shown in Table 2.

**Table 2:** List of academic and governmental databases searched in the systematic literature review, with internet links (accessed July 2022).

Database	Link	Inclusion in literature review?	Reasoning for inclusion or exclusion
**ProQuest–Aquatic Sciences and Fisheries Abstracts (ASFA)**	https://www.proquest.com/asfa/	test in initial queries	Should be covered by WoS
**PubMed**	https://pubmed.ncbi.nlm.nih.gov/	no	Covered by WoS
**Scopus**	https://www.scopus.com/search/	test in initial queries	Should be covered by WoS
**Cochrane Library**	https://www.cochranelibrary.com/	no	Covered by WoS
**Web of Science (Core Collection)**	https://www.webofscience.com/wos/woscc/	yes	Most comprehensive directory–covers all other databases. Can save searches, and provides plugin functions to download references to reference manager–great for transparency
**JSTOR**	https://www.jstor.org/	no	Covered by WoS
**GreenFILE**	https://web.p.ebscohost.com/ehost/search/	no	Covered by WoS
**ScienceDirect**	https://www.sciencedirect.com/search	no	Covered by WoS
**Federal Registration of Legislation (Australia)**	https://www.legislation.gov.au/	yes	Federal and State Legislation for Australia
**State and Territory Legislation**	https://www.aph.gov.au/about_parliament/parliamentary_departments/parliamentary_library/browse_by_topic/auslaw	no	Covered by ‘legislation.gov.au’
**GovPubs**	https://www.nla.gov.au/apps/govpubs/	yes	Government Publications Database

Australia has databases of both Commonwealth (Federal) and State Government legislation that will be systematically searched using terms in Table 3. Search terms have been adapted from a previously published study looking at the drivers of protected area development in Australia (REF: S. Hernandez, C. Benham, R.L. Millier, M. Sheaves, S. Duce (2021) What drives modern protected area establishment in Australia? *Conservation Science and Practice*. 3:e501. DOI: doi.org/10.111/csp2.501). The search terms will be modified from this paper to include ‘marine protected area’ and ‘partially protected area’ as relevant search terms. These same search terms will be used in the Australian government publication database–GovPub—to include departmental reports where they have been made available to the public (see Table 2).

**Table 3:** The following BOOLEAN search terms will be used in the study, with their function displayed on the right. Included in the search is a filter to limit the scientific literature to the previous 11 years (2011–2022).

	Objectives
(TS = ((marine protected areas) OR (partially protected marine protected areas) OR (partially protected MPA) OR (part* protec* marine protected area*) OR (partial protection) OR (partial protec* in MPA) OR (partially protected area) OR (Sea Country) OR (Indigenous Protection Area) OR (exclusion zone) OR (exclusion zon*) OR (nursery area) OR (exclusion area) OR (net free zone) OR (net-free zon*) OR (trawl exclusion zone) OR (pipeline protection zone) OR (research area) OR (research zone) OR (research closure) OR (research area closure) OR (multiple use zone) OR (multi* use zon*) OR (Australi* Marine Protected Areas) OR (Australi* MPAs) OR (Australi* partially protected areas) OR (Australi* parti* protec* area*) OR (Australi* fisheries management) OR (Australi* sustainable fisheries management) OR (triple bottom line) OR (soci* object*) OR (econ* object*) OR (biod* object*) OR (soci* goal*) OR (cons* object*) OR (cons* goal*) OR (econ* goal*) OR (biod* goal*) OR (cultural* object*) OR (cultural* goal*))) AND TS = ((Aus*) OR (Austral*)) and Article or Proceeding Paper or Review Article or Early Access or Correction (Document Types) and Environmental Sciences or Ecology or Marine Freshwater Biology or Environmental Studies or Fisheries or Economics or Water Resources or Oceanography or Biodiversity Conservation or Multidisciplinary Sciences or Geosciences Multidisciplinary or Law or Management or Green Sustainable Science Technology or Engineering Environmental or Geography or History or Sociology or Hospitality Leisure Sport Tourism or Geography Physical or Zoology or International Relations or Agronomy or Remote Sensing or Mathematics Applied or Evolutionary Biology or Parasitology or Social Issues or Biology or Behavioral Sciences or Ethics or Cultural Studies or Agricultural Economics Policy or Mathematics or Geology or Mathematical Computational Biology or Engineering Ocean or History Of Social Sciences or Engineering Marine (Web of Science Categories) and AUSTRALIA (Countries/Regions) and English (Languages)	1. Scientific peer-reviewed literature search 2. Specifies the broad scope relevant to primary and secondary research questions 3. Limits to Australia and English-language 4. Limits fields of study to exclude fields judged to be not relevant to the review (i.e. medical / oncology fields). 5. Limits time span to previous 10 years of research in Australia, however this may be revised if insufficient studies are found.
“biodiversity” OR “reserves” OR “protected areas” OR “conservation” OR “marine protected areas” OR “partially protected areas” AND “Australia” OR “Australian Capital Territory” OR “ACT” OR “Northern Territory” OR “NT” OR “New South Wales” OR “NSW” OR “Queensland” OR “Qld” OR “Tasmania” OR “Tas” OR “South Australia” OR “SA” OR “Western Australia” OR “WA” OR “Victoria” OR “Vic.”	Search terms for legislation databases Search terms for Australian Government publication database GovPub

The search will be conducted in English, which risks missing valuable literature and research conducted in other languages and could contribute to known geographical biases in research [25] and create barriers to disseminating research internationally. However, as the scope of the literature review is limited to Australia, and Australian territories, we are confident that few studies on Australian PPAs will be missed. We will endeavour to publish and disseminate results as widely as possible, within appropriate international author collaborations and networks.

All literature searches will be limited to the previous 11 years (2011–2022) to ensure we are covering the most recent studies in this field. This timeframe is of particular interest because many OECMs, PPAs and MPAs have recently been implemented in these years in response to the global Aichi Target 11 (10% protection by 2020), and additionally it gives a reasonable timeframe to assess the impacts of the first impacts of PPAs and MPAs implemented in this period, and the early 2000s. This period will also ensure studies reflect the rapidly changing climate in Australian waters. Importantly, targeted grey literature from research conducted within government departments, such as the National Environmental Science Program (NESP) will also be included in the review.

### Search terms

Search terms will be validated by identifying multiple benchmark studies in the field and identifying whether these studies are returned based on selected search terms.

### Article screening

We will follow the ROSES flow diagram to screen articles, apply consistency checks and ensure eligibility criteria are met [22]. All articles returned by the search will be screened by the lead author (GACP), who will conduct title, abstract screening, and decide on ‘inclusion‘, ‘exclusion’ or ‘further advice’. Title and abstract screening will be conducted using the R package ‘*Revtools*’ to remove duplicates and ensure that ‘included’ papers contain relevant material to the literature review ([26]. *Revtools* is an R package that supports article screening in systematic reviews–the software imports, deduplicates and screens articles for relevance. A list of all publications will be kept, including both articles retained for review, and those removed, ensuring full transparency and opportunities for constructive feedback. Results from all Revtools screening, including R code will also be made available for others to use.

Final lists of all articles in the initial search, those to be included and those to be excluded will be available for the entire project team for any additional feedback or categorisation prior to the review, according to the eligibility criteria. Once the final list of articles is ready for the review, a randomised subset of included articles will be screened for consistency, to ensure only relevant papers are included in the review.

### Eligibility criteria

To ensure that articles included in the review are sufficiently relevant to the literature review, the following eligibility criteria will be applied to articles prior to review.

#### Eligible subjects and living populations

Articles will only be considered if they are related to marine or inshore (brackish) environments (i.e., not freshwater rivers or lakes). Freshwater areas will be excluded as these are covered under different legislation and independent IUCN goals for conservation protection. Articles will be included if they relate to Australian marine or inshore environments (Australian Federal, Commonwealth waters, and State and Territory waters). This includes Australia’s remote territories (e.g. Macquarie Island, Cocos (Keeling) Islands, Christmas Island), but excludes Antarctica as legislation is non-binding in this region and includes geo-political complexities surrounding the Convention for the Conservation of Antarctic Marine Living Resources (CCAMLR).

#### Eligible interventions

Articles that relate to the implementation or planned implementation of marine protected areas of any kind or definition within Australian waters will be considered.

#### Eligible outcomes

Any outcomes from eligible interventions will be considered.

#### Eligible study types

Studies will be considered eligible if they relate to real-world marine protected areas in Australian waters (rather than theoretical simulations or extrapolations of studies in other areas of the world to Australia). Studies that include components of mathematical modelling as applied to real-world scenarios will be included. Peer-reviewed scientific publications published at any point in time (as long as they are within the appropriate databases) will be included. Legislation, policy documents, and agreements or management plans organised in collaboration with Traditional Owners will be considered, if information is in publicly available databases.

### Study validity assessment

As all literature within the review has already undergone a form of review (either prior to publication in a peer-reviewed journal article, as part of a legislative or policy writing process, or prior to publishing from a government department), the validity of studies will not be assessed in this review. However, critical judgement will be reserved as to the appropriate weighting of results from articles.

### Data extraction

Data will be extracted from each article selected for detailed review. Data here refers to documented information addressing the main questions for the literature review (as opposed to data from specific studies). For example, how a study defined ‘partially protected areas’. Data will be stored online. and accessible to the author team. Articles will be reviewed by the author team using a cross-validation system to ensure objectivity in data extraction (i.e. multiple authors review the same papers). We will catalogue all references used in reference software (Mendeley).

#### Metadata extracted

The following metadata will be extracted from all peer-reviewed scientific papers and grey literature (including policy documents). These bullet points will be used as column headings in a spreadsheet. Not all headings will be able to be populated by every article screened due to the content of the document being reviewed.

- Person screening the document
- Article DOI
- Article type (e.g. peer-reviewed publication or legislative document)
- Article title
- Article authors
- State / Territory / area of Australia the study or legislation/policy is based
- Protection classification of MPAs within article or legislation (based on The MPA Guide [27]
- Aspects of MPA studied if relevant (e.g. social consequences of implementing an MPA)
- Fisheries management and/or fisheries relevant regulations within MPA?
- Parks or conservation relevant regulations within MPA?
- Indigenous agreements or management regulations within MPA?
- Monitoring plan in place?
- What is being monitored? And how?
- Analyses of monitoring data?
- Outcome of monitoring / MPA implementation?
- This relates to whether the MPA is delivering on the goals it has specified
- Is MPA listed and visible on https://mpatlas.org/?
- Have there been any major changes to management of the PPA over time (if the PPA has been established for more than 10 years (i.e. prior to 2011))?
  ○ What are these changes?

### Data synthesis

A key output from the review will be a comprehensive overview of the range and scope of PPAs in Australian waters. A table will provide the goals for those areas, and whether monitoring plans are effectively capturing relevant data to meet those goals. Another key output will be a synthesis of the relevant legislation and policies covering PPAs in Australian marine waters, and whether there is any overlap between fisheries and conservation-led management plans.

Coverage of PPAs in Australia will be represented visually by generating detailed maps covering all types of protected marine areas, including descriptive statistics. If multiple studies have been conducted on a specific PPA, a meta-analysis of data within those articles may be warranted, if the results will help to answer either a primary or secondary research question.

Synthesis of management options and measured outcomes from PPAs will be provided as a key output. Future management options or policy recommendations will also be provided if these are identified as being relevant.

### Risk of publication bias

Search terms have deliberately been designed to be broad, without the use of potentially bias-inducing terms such as, for example, ‘increasing’ or ‘decreasing’, ‘beneficial’ or ‘ineffectual’[28,29]. This will reduce the risk of including potentially biased studies. The study will also look at both peer-reviewed literature and ‘grey’ literature, such as government reports, policies, and legislation to avoid biasing the literature towards specific scientific disciplines. The search terms are deliberately broad to encompass literature from conservation as well as fisheries-focussed literature. The project has also appointed a Steering Committee that will consist of members from a diverse range of backgrounds, including Indigenous, commercial, recreational, and conservation-based marine resource users. The steering committee will form an integral and influential part of this project, providing continuous feedback on the review prior to final publication.

### Procedural independence

Members of the project team and Steering Committee work within research areas related to fisheries management, marine ecology and conservation. It is thus inevitable that studies will be included that have been written or performed by members of both groups. For transparency, a list of all publications in the search, those included, those excluded, and reasoning for all decisions will be provided as metadata to accompany the review when published.

### Confidence in cumulative evidence

The systematic literature review protocol presented here does not include quantitative data analyses. However, the review will be an unbiased qualitative representation of the current literature surrounding an important marine resource management question according to peer-reviewed publications, government reports, policy and legislation. We will assume that peer-reviewed literature has undergone substantial peer-review, and will evaluate the evidence from these papers in an objective manner (as described in earlier sections). This will ensure confidence in the cumulative evidence that the review provides.

### Ethics and dissemination

Ethics are not required for a systematic literature review of published literature. No interviews will be conducted regarding the research questions. Dissemination of the results of the systematic literature review will be via peer-reviewed publication, in milestone reports for the FRDC Reports, and in stakeholder meetings, scientific conferences, and workshops with marine resource managers and users.

## Discussion

This study has been conceived, designed, and will be implemented in response to a need to better understand the potential implications of partially protected areas (PPAs) in Australia’s marine environment. The study will provide information for ecological, social, and economic perspectives. Therefore, the Steering Committee for the project, and the research team are comprised of researchers and stakeholders from a broad range of disciplines and backgrounds. The methods, narrative and qualitative analyses that will be conducted in this systematic review will provide tangible outcomes for marine resource managers in Australia, and further afield. Any policy and management recommendations from this review will be presented to relevant marine resource managers in a manner that will allow for constructive discussion about the future and possible applications of PPAs in Australia.

## Supporting information

S1 Checklist PRISMA-P 2015 checklist. (DOCX)
